# Report on the Influence of Light on the Growth of Plants

**Published:** 1847-12

**Authors:** R. Hunt


*Report on the Influence of Light on the Growth of Plants.* By R. Hunt.—The author confirms the conclusions that seeds will not germinate under the influence of light separated from the chemical principle with which it is associated in the sunbeam; that germination being effected, and the first leaves formed, light—the luminous rays become essential to the plant to enable it to secrete the carbon obtained from the carbonic acid of the atmosphere; and that the increased action of the heat rays is essential to insure the production of the reproductive elements of vegetable life. It is found that the chemical principle of the solar rays is more active, relatively to heat and light, during the spring than at any other period of the year; that as summer advances, this power diminishes, and luminous force increases, while with the autumn both light and actinism are subdued, but the calorific radiations increased. Thus we find the conditions of the light of the seasons varying to suit the necessities of vegetable life. The production of chlorophyl, or the coloring matter of the leaves, was shown to be due to the joint action of light and actinism—the first being necessary to effect the secretion of the carbon, and the latter for the oxidation of this deposited carbon.—*Transactions of Brit. Association.—Jour. Franklin Institute.*

